# Using 24-h Heart Rate Variability to Investigate the Sleep Quality and Depression Symptoms of Medical Students

**DOI:** 10.3389/fpsyt.2021.781673

**Published:** 2022-01-04

**Authors:** Xiansheng Guo, Tiehong Su, Haoran Xiao, Rong Xiao, Zhongju Xiao

**Affiliations:** ^1^Department of Psychology, School of Public Health, Southern Medical University, Guangzhou, China; ^2^Department of Physiology, School of Basic Medical Sciences, Southern Medical University, Guangzhou, China; ^3^Department of Clinical Electrophysiology, The Seventh Affiliated Hospital, Southern Medical University, Foshan, China

**Keywords:** sleep quality, depressive symptoms, positive and negative affects, 24-h heart rate variability, medical students

## Abstract

There have been numerous studies on the relationship between sleep and depression, as well as the relationship between sleep and depression, and heart rate variability (HRV), respectively. Even so, few studies have combined 24-h HRV analysis to study sleep quality and depressive symptoms. The purpose of this cross-sectional study was to investigate the relationship between depressed symptoms, sleep quality, and 24-h HRV in medical students. The particiants were all students at a medical university in Guangdong province, China. A total of 74 college students participated. They were asked to complete a questionnaire that included the Pittsburgh Sleep Quality Index (PSQI), the Beck Depression Inventory-II (BDI-II), the Positive and Negative Affect Scale (PANAS), and 24-h ECG monitoring. The results showed that 41.7% of the medical students had poor sleep quality, with higher levels of depressive symptoms and more negative emotions, and there was no difference in 24-h HRV indices between the low PSQI group and the high one. Correlation analysis showed that there was a significant relationship between sleep quality and depressive symptoms (*r* = 0.617), but the relationship between 24-h HRV indices and PSQI global scores, BDI scores were not significant. However, the correlation analysis of PSQI components and 24-h HRV showed that sleep disturbance was significantly negatively correlated with SDNN and LF in waking period (*r* = −0.285, −0.235), and with SDNN in sleeping period (*r* = −0.317). In general, the sleep disturbance in PSQI components can sensitively reflect the relationship between sleep quality and 24-h HRV of medical students. Individuals with higher sleep disturance may have lower SDNN during awake period and bedtime period, and lower LF in awake period. Twenty-four hour HRV has certain application value in clinical sleep quality monitoring, and its sensitivity and specificity in clinical application and daily life are still worth further investigation.

## Introduction

Heart rate variability (HRV) is an important indicator of autonomic nervous system activity, and has been paid more and more attention for its objective, convenient, non-invasive, and sensitive characteristics. Heart rate variability is the fluctuation in time between successive heartbeats and is defined by interbeat intervals (1). Heart rate variability analysis methods mainly include time domain and frequency domain. Low HRV is thought to be a marker for a number of mental health problems, including the severity of psychiatric disorders such as insomnia, depression, and anxiety (2–10). Insomnia and depression were found to be independent risk factors for cardiovascular disease morbidity and mortality (11–13), suggesting that patients with depression and insomnia may have cardiac autonomic dysfunction. Poor sleep quality and mental disorders such as depression and anxiety disorder can be reflected by HRV (11–15). In terms of time domain indices, standard deviation of the NN (SDNN), root mean square of R–R-intervals, (RMSSD) or the ratio of adjacent R–R intervals to total R–R intervals (pNN50) was lower than that of normal control group. And in terms of frequency domain indices, the low frequency components (LF) and high frequency components (HF) are lower, while the LF/HF components are higher, among which the decrease of HF components is the most common. The parasympathetic nervous system is the branch of the autonomic nervous system responsible for critical restorative processes like “resting and digesting” (16). The effective functioning of this system has been associated with stronger phasic activity of vagus nerve efferent activity to the sino-atrial node of the heart, often termed *cardiac vagal control* (CVC).

Many studies have linked CVC to sleep. Although higher CVC during wakefulness (CVC_wake_) (17–19) and higher CVC during sleep (CVC_sleep_) (10, 20, 21) are both associated with higher sleep quality, some studies have found no significant relationship between CVC_wake_ and CVC_sleep_, or found that CVC_wake_ but not CVC_sleep_ is associated with better sleep quality (21, 22). The relationship between CVC_wake_, CVC_sleep_ and sleep quality remains to be further verified. Cardiac vagal control during wakefulness and sleep may be related to different sleep processes, and both of them may be predictors of sleep quality. Sleep is essential for normal brain function and mental health (23, 24). The maintenance and promotion of health is important to college student success. The study has shown that the phenomenon of sleep disturbance is not only common among Chinese medical students, but also higher than non-medical students and the general population (25), which may have a bad impact on their academic performance, physical and mental health, and quality of life (26). A recent meta-analysis of 43 studies involving a total of 18,619 students from 13 countries found that lack of sleep is a common problem among medical students, and they sleep an average of 6.3 h, <7 h of sleep advice, with 33 studies finding that more than half of the medical students report poor sleep quality (27). Sleep disorders can manifest as complaints of insufficient sleep, excessive sleep, or abnormal movement during sleep (28). Studies have shown that sleep disorders can lead to decreased immunity, decreased life adaptability, anxiety, depression, and other problems, and even cause a variety of physical diseases (29). College students are often characterized by irregular sleep time, insufficient sleep, and poor sleep quality (30, 31), and the sleep quality of medical students is still not optimistic (25, 32). Sleep disturbance is a risk factor for depression (33), and sleep quality index predicts future depression, anxiety, and stress scores in college students at both baseline and follow-up (34). Some research even reported that a complex bidirectional relationship between depression and sleep (35, 36). Sleep disturbance is receiving increasing attention as a public health issue.

Finally, The recordings and analysis of HRV can be as short as a few minutes or as long as 24 h. Each has its own advantages and cannot be replaced by the other. Long-term HRV recordings predict health outcomes heart attack, stroke, and all-cause mortality. Though the prognostic value of long-term HRV assessment, it has not been broadly integrated into mainstream medical care or personal health monitoring (1). Although there has been much research on the relationship between sleep problems and depression, a review of the literature found that there have been few current studies on the relationship between depression and sleep disorders combined with 24-h HRV analysis. The sensitivity and specificity of 24-h HRV in normal subjects and clinical samples required additional investigations, and the findings of previous studies on the relationship between CVC and sleep quality are extremely disparate. The purpose of this study was to explore the relationship between sleep quality, depressive symptoms and 24-h HRV among medical students. Our research hypothesis as follows: First, varied sleep quality can be reflected in 24-h HRV, and there are significant differences in HRV between groups with varying sleep quality. Second, CVC has a significant positive relationship with sleep quality in both the waking and sleeping phases.

## Materials and Methods

### Participants and Procedure

Participants in this cross-sectional study were recruited from a medical university in Guangzhou, Guangdong, China, using a website link's recruiting information and experimental recruitment ads. The study was carried out from October 2020 to July 2021, and we received response from 80 potential participants. Our inclusion criteria were as follows: First, no history of mental illness, such as diagnosed depression, anxiety, or other mental diseases. Second, no history of heart illness, such as coronary heart disease, arrhythmia, cardiomyopathy, and so on. Third, no long-term smoking or drinking behaviors. A total of 76 participants were recruited for this cross-sectional study based on initial screening results.

Participants then arrived at the lab on time to complete a questionnaire containing the Pittsburgh Sleep Quality Index (PSQI), Beck Depression Inventory -II, BDI-II), The Positive and Negative Affect Scale (PANAS), and wear a dynamic electrocardiograph to complete a 24-h dynamic electrocardiogram examination in school, with sensors connecting the electrocardiograph to the individual's sternum in the center. During menstruation, female participants were not subjected to 24-h ECG monitoring. Four participants were excluded [one participant did not complete a 24-h ECG monitoring, one participant had a history of anxiety disorder, and two participants detected ventricular premature beat (VPB)]. Finally, data from 72 participants (31 males and 41 females, aged 19–34 years, *M* = 23.90, *SD* = 2.39) were included in the analysis, who were divided into low PSQI group and high PSQI group based on PSQI total scores. The study was approved by the ethics committee of Southern Medical University and all participants provided informed consent.

### Measures

#### The Pittsburgh Sleep Quality Index

Subjective sleep quality was evaluated by the Chinese version of the PSQI, a widely used, self-report questionnaire that assesses sleep quality during the previous month (37). The Chinese version of the PSQI consists of 19 self-rated questions and five questions rated by the bed partner or roommate. The 19 items are grouped into seven component scores, each weighted equally on a 0–3 scale. The seven components are subjective sleep quality, sleep latency, sleep duration, habitual sleep efficiency, sleep disturbances, use of sleeping medications, and daytime dysfunction. The component scores are summed to yield a PSQI global score (range: 0–21). A score above five would indicate poor sleep quality and higher scores indicate worse sleep quality (37). The Chinese version of the PSQI had satisfactory psychometric properties in previous studies (38), which is an effective tool to investigate and screen the sleep quality of medical students in China (39). In our research, the Cronbach's alpha was 0.774.

#### Beck Depression Inventory-II

The self-reported Beck Depression Inventory (BDI-II) (40, 41) was used to assess depressive symptoms among medical students. Beck Depression Inventory-II consists of 21 items, each of which is scored from 0 to 3. Responses were summed up to yield the total score, which could range from 0 to 63, with higher scores meaning higher levels of depressive symptoms (42). The Chinese version has good reliability and validity, with Cronbach's alpha 0.94 (43).

#### The Positive and Negative Affect Scale

The PANAS (44) consist of two 10-item mood scales and was developed to provide brief measures of PA and NA. Respondents are asked to rate the extent to which they have experienced each particular emotion within a specified time period, with reference to a five-point scale. Each adjective was rated on a scale from 1 = very slightly to 5 = extremely. A number of different time-frames have been used with the PANAS, but in the current study the time-frame adopted was “during the past week.” In our research, the Cronbach's alpha was 0.925.

#### 24-h ECG Monitoring

Holter recordings (Mobio^®^ Portable Recorder, Chengdu Synwing Technology Co., Ltd., Chengdu, China) were used to obtain continuous 24-h ECG data. During testing, participants were asked to maintain their daily activities and to avoid drinking alcoholic beverages. Participants were asked to record the time when they slept and when they woke up. The bedtime ECG was defined as the recording obtained from the time at which subjects went to bed until the time they got out of bed, and the awake ECG as that obtained during the rest of day (10). All the subjects underwent ECG monitoring at school. ECG analytics software was used to process and analyze the collected data, and the time domain indices and frequency domain indices were obtained. Time domain measures of HRV include the mean heart rate and standard deviation of the normal interbeat intervals (SDNN), the root mean square successive difference (RMSSD) between adjacent normal interbeat intervals, and the percentage of adjacent intervals that varied by >50 ms (pNN50). Standard deviation of the normal interbeat interval is indicated to reflect overall HRV and RMSSD as well as pNN50 reflect parasympathetic activity (6). Frequency domain of measures HRV include high-frequency power (HF: 0.15–0.40 Hz), low-frequency power (LF: 0.04–0.15 Hz), and LF/HF ratio. Low-frequency power is thought to be modulated by both sympathetic and parasympathetic activities, whereas HF power is mainly modulated by parasympathetic activity. The LF/HF ratio was computed as a measure of the sympathovagal balance toward sympathetic activity (45, 46).

### Data Analysis

Descriptive analysis and difference test between two independent samples were performed using SPSS 22.0. The collected demographic data, sleep quality index, depressive symptoms, positive and negative emotions, and 24-h HRV were analyzed by descriptive analysis. Since the variables in our study did not conform to normal distribution, Mann-Whitney U-test was used to test inter-group differences in depressive symptoms, HRV, as well as positive and negative emotions, as well as differences in HRV during wakefulness and sleep periods. Spearman rank correlation analysis was used to test the relationship between PSQI, depressive symptoms, and HRV, and to test the relationship between sleep quality index, depressive symptoms, and HRV during wakefulness and bedtime period, *p* < 0.05 was used to confirm statistical significance.

## Results

### Demographic Data of the Total Sample and Descriptive Statisics

The age, sex ratio, height, weight, BMI [BMI (kg/m^2^) = weight (kg)/Height^2^ (m)], PSQI global scores, depressive symptoms, positive and negative emotions, and all 24-h HRV indices of the total study samples are shown in Table 1.

**Table 1:** Demographic data of the total sample.

**Variable**		***Mean***	***SD***
Age (years)		23.90	2.39
Male/Female	31/41		
Height (m)		1.65	0.08
Weight (kg)		56.53	8.90
Heart rate (beats/min)		74.57	6.77
BMI (kg/m^2^)		20.65	2.30
PSQI global score		5.29	2.84
BDI-II score		8.26	8.61
**PANAS**			
Positive		30.07	6.57
Negative		21.39	7.16
**HRV**			
SDNN		170.09	34.74
RMSSD		45.02	14.93
pNN50		20.20	10.33
LF		1105.63	563.60
HF		849.81	581.01
LF/HF		1.60	0.89

*PANAS, positive and negative affect scale; HRV, heart rate variability*.

### Differences in Depressive Symptoms, 24-h HRV and Positive and Negative Emotions Between Different Sleep Quality Groups

As shown in Table 2, the study divided the sample into two groups of participants, 42 of the 72 participants had good sleep quality (PSQI global score ≤ 5; Age, 24.07 ± 2.65), accounting for 58.3% of the total samples. Thirty participants had poor sleep quality (PSQI total score >5 points; Age, 23.67 ± 1.99), accounting for 41.7% of the total samples. There were no differences between the two groups in terms of age, gender ratio, BMI, or heart rate.There were significant differences in PSQI global scores (3.48 ± 1.45 vs. 7.83 ± 2.29, *p* < 0.001) and BDI-II scores (5.02 ± 4.51 vs. 12.80 ± 10.79, *p* < 0.001) between the low PSQI group and the high PSQI group. At the same time, the group with low PSQI experienced more positive emotions (32.33 ± 6.56 vs. 26.90 ± 5.32, *p* = 0.001) and less negative emotions (19.21 ± 5.23 vs. 24.43 ± 8.12, *p* = 0.003) in the recent period than the group with high PSQI.

**Table 2:** Differences of depressive symptoms, heart rate variability and positive and negative emotions in sleep quality group.

**Variable**	**Low PSQI group** **(***n*** = 42)**	**High PSQI group** **(***n*** = 30)**	***p*** **^a^**
Age	24.07 ± 2.64	23.67 ± 2.00	0.719
Gender (male/female)	20/22	11/19	0.358
Height (m)	1.66 ± 0.08	1.64 ± 0.07	0.552
Weight (kg)	56.80 ± 8.39	56.15 ± 9.70	0.652
Heart rate (beats/min)	74.31 ± 6.84	74.93 ± 6.76	0.744
BMI	20.66 ± 2.19	20.64 ± 2.48	0.964
PSQI global score	3.48 ± 1.45	7.83 ± 2.29	0.000
BDI-II score	5.02 ± 4.51	12.80 ± 10.79	0.000
**PANAS**			
Positive	32.33 ± 6.48	26.90 ± 5.32	0.001
Negative	19.21 ± 5.23	24.43 ± 8.12	0.003
**HRV**			
SDNN (ms)	172.34 ± 33.83	166.96 ± 36.33	0.486
RMSSD (ms)	44.95 ± 14.83	45.12 ± 15.32	0.977
pNN50 (%)	20.21 ± 10.28	20.19 ± 10.57	0.991
LF (ms^2^)	1130.27 ± 608.21	1071.13 ± 502.58	0.486
HF (ms^2^)	823.16 ± 566.95	887.11 ± 607.91	0.623
LF/HF	1.59 ± 0.75	1.62 ± 1.07	0.652

a *Comparison between the two groups (non-paried): continuous variable (Mann-Whitney U-test). Low PSQI group indicated that the scores were below 5 and good sleep quality; High PSQI group indicated that the scores were above 5 and poor sleep quality. PANAS refers to the Positive and Negative Affect Scale. HRV refers to heart rate variability. Mean values (± SD) for the indices of each group*.

### Comparison of Heart Rate Variability of Different Sleep Quality During Awake and Bedtime Period

The overall mean and standard deviation of all HRV indices, and the mean and standard deviation of HRV indices during awake period and bedtime period are shown in Table 3. Mann-Whitney U-test was used to test whether there was a difference in HRV between the low PSQI group and the high PSQI group during awake period and bedtime period. The results showed that there was no significant statistical difference in HRV indicators between the group with low PSQI and the group with high PSQI (*p* > 0.05).

**Table 3:** Heart rate variability characteristics and differences during awake and bedtime period.

**Variable**	**Mean** **±** **SD**			***p*** **^a^**
	**Total (***n*** = 72)**	**Low PSQI group (***n*** = 42)**	**High PSQI group (***n*** = 30)**	
**Awake period**				
Time domain				
SDNN (ms)	123.11 ± 29.71	125.09 ± 30.15	120.34 ± 29.36	0.398
RMSSD (ms)	36.52 ± 13.08	37.17 ± 13.90	35.62 ± 12.02	0.583
pNN50 (%)	14.50 ± 9.44	14.97 ± 9.69	13.85 ± 9.19	0.545
Frequency domain				
LF (ms^2^)	971.36 ± 392.77	1004.30 ± 407.73	925.25 ± 372.69	0.253
HF (ms^2^)	551.85 ± 449.87	554.94 ± 465.32	547.52 ± 435.14	0.936
LF/HF	2.39 ± 1.35	2.38 ± 1.11	2.39 ± 1.65	0.530
**Bedtime period**				
Time domain				
SDNN (ms)	116.21 ± 30.99	117.01 ± 29.29	115.10 ± 33.69	0.553
RMSSD (ms)	63.86 ± 24.83	63.38 ± 22.62	64.54 ± 28.03	0.950
pNN50 (%)	37.90 ± 16.44	37.22 ± 16.87	38.85 ± 16.05	0.564
Frequency domain				
LF (ms^2^)	1312.82 ± 863.15	1215.85 ± 573.19	1448.59 ± 1151.89	0.855
HF (ms^2^)	1614.01 ± 1320.89	1608.75 ± 1392.53	1621.3787 ± 1236.93	0.623
LF/HF	1.08 ± 0.72	1.03 ± 0.52	1.15 ± 0.95	0.855

a *Comparison between the two groups (non-paried): continuous variable (Mann-Whitney U-test). Low PSQI group indicated that the scores were below 5 and good sleep quality; High PSQI group indicated that the scores were above 5 and poor sleep quality. HRV refers heart rate variability. Mean values (± SD) for the indices of each group*.

### Correlation Analysis of Sleep Quality, Depressive Symptoms and 24-h HRV

The mean scores for the PSQI global scores, BDI-II scores, SDNN, RMSSD, pNN50, LF, HF, and LF/HF were 5.29 (*SD* = 2.84), 8.26 (*SD* = 8.61), 170.09 (*SD* = 34.74), 45.02 (*SD* = 14.93), 20.20 (*SD* = 10.33), 1105.63 (*SD* = 563.60), 849.81 (*SD* = 581.01), 1.60 (*SD* = 0.89), respectively. Spearman correlations were used to explore the relationship between sleep quality, depressive symptoms, and 24-h HRV, as shown in Table 4. The results found that there was a significant correlation between PSQI global scores and depressive symptoms (*r* = 0.617, *p* < 0.01), while there was no significant correlation between the indicators of 24-h HRV and the first two, so further analysis could not be carried out.

**Table 4:** Descriptive statistics and correlation analysis for PSQI global score, BDI-II score, and 24-h HRV.

**Variable**	***M* ±*SD***	**1**	**2**	**3**	**4**	**5**	**6**	**7**	**8**
1. PSQI global scores	5.29 ± 2.84	1							
2. BDI-II socres	8.26 ± 8.61	0.617**	1						
3. SDNN	170.09 ± 34.74	−0.122	−0.092	1					
4. RMSSD	45.02 ± 14.93	0.002	0.014	0.591**	1				
5. pNN50	20.20 ± 10.33	0.003	0.039	0.523**	0.970**	1			
6. LF	1105.63 ± 563.60	−0.090	0.010	0.606**	0.665**	0.576**	1		
7. HF	849.81 ± 581.01	0.068	0.036	0.582**	0.952**	0.920**	0.601**	1	
8. LF/HF	1.60 ± 0.89	−0.115	−0.021	−0.255*	−0.635**	−0.655**	0.034	−0.725**	1

* *p < 0.05*,

** *p < 0.01*.

### Correlation Analysis of Sleep Quality, Depressive Symptoms and HRV During Awake Period

Table 5 shows the mean and standard deviation of PSQI dimensions, PSQI global scores, BDI-II, and HRV indicators during awake period. Spearman correlations were used to explore the relationship between sleep quality, depressive symptoms, and HRV during awake period.We found that the score of Sleep disturbance is negatively correlated with SDNN and LF (*r*_*SDNN*_ = −0.285, *r*_*LF*_ = −0.239, *p* < 0.05), and positively correlated with BDI-II (*r* = 0.372, *p* < 0.01) during awake period. However, we did not find a significant correlation between LF, SDNN and BDI-II scores during awake period (*r* = 0.040, −0.021, *p* = 0.740, 0.864).

**Table 5:** Descriptive statistics and correlations for primary PSQI indices of sleep quality, BDI-II score, and HRV during awake period.

**Variable**	***M* ±*SD***	**1**	**2**	**3**	**4**	**5**	**6**	**7**	**8**	**9**	**10**	**11**	**12**	**13**	**14**	**15**
**PSQI component scores**																
1. Subjective sleep quality	1.04 ± 0.72	1														
2. Sleep latency	0.85 ± 0.87	0.554**	1													
3. Sleep duration	0.79 ± 0.50	0.269*	0.158	1												
4. Habitual sleep efficiency	0.10 ± 0.34	0.157	0.091	0.121	1											
5. Sleep disturbance	0.96 ± 0.49	0.374**	0.339**	0.173	0.137	1										
6. Use of sleeping medication	0.14 ± 0.51	0.273*	0.341**	0.300*	0.095	0.230	1									
7. Daytime dysfunction	1.42 ± 0.95	0.511**	0.398**	0.204	0.187	0.330**	0.389**	1								
8. PSQI global scores	5.29 ± 2.84	0.769**	0.763**	0.396**	0.259*	0.535**	0.454**	0.774**	1							
9. BDI-II score	8.26 ± 8.61	0.555**	0.383**	0.191	0.147	0.372**	0.288*	0.663**	0.617**	1						
10. SDNN	123.11 ± 29.71	−0.046	−0.118	−0.106	0.032	−0.285*	−0.099	0.018	−0.127	0.040	1					
11. RMSSD	36.52 ± 13.08	−0.022	−0.016	−0.002	−0.039	−0.112	−0.112	−0.011	−0.035	0.040	0.667**	1				
12. pNN50	14.50 ± 9.44	−0.059	−0.024	−0.008	−0.042	−0.128	−0.100	−0.021	−0.057	0.027	0.645**	0.982**	1			
13. LF	971.36 ± 392.77	−0.093	−0.086	−0.082	0.085	−0.239*	−0.146	−0.026	−0.136	−0.021	0.608**	0.685**	0.665**	1		
14. HF	551.85 ± 449.87	−0.029	0.101	−0.017	0.030	−0.043	−0.128	−0.001	0.041	0.053	0.573**	0.930**	0.906**	0.680**	1	
15. LF/HF	2.39 ± 1.35	−0.037	−0.202	−0.034	0.106	−0.078	0.088	0.064	−0.119	−0.042	−0.308**	−0.694**	−0.670**	−0.114	−0.757**	1

* *p < 0.05*,

** *p < 0.01*.

### Correlation Analysis of Sleep Quality, Depressive Symptoms and HRV During Bedtime Period

Table 6 shows the mean and standard deviation of PSQI dimensions, PSQI global scores, BDI-II scores, and HRV indicators during bedtime period. We only found a negative correlation between Sleep disturbance scores and SDNN (*r* = −0.317, *p* < 0.01) during bedtime period, and a positive correlation between BDI-II scores (*r* = 0.372, *p* < 0.01). However, we did not find a significant correlation between SDNN and BDI-II scores during bedtime period (*r* = −0.108, *p* = 0.37).

**Table 6:** Descriptive statistics and correlations for primary PSQI indices of sleep quality, BDI-II score, and HRV during bedtime period.

**Variable**	***M* ±*SD***	**1**	**2**	**3**	**4**	**5**	**6**	**7**	**8**	**9**	**10**	**11**	**12**	**13**	**14**	**15**
**PSQI component scores**																
1. Subjective sleep quality	1.04 ± 0.72	1														
2. Sleep latency	0.85 ± 0.87	0.554**	1													
3. Sleep duration	0.79 ± 0.50	0.269*	0.158	1												
4. Habitual sleep efficiency	0.10 ± 0.34	0.157	0.091	0.121	1											
5. Sleep disturbance	0.96 ± 0.49	0.374**	0.339**	0.173	0.137	1										
6. Use of sleeping medication	0.14 ± 0.51	0.273*	0.341**	0.300*	0.095	0.230	1									
7. Daytime dysfunction	1.42 ± 0.95	0.511**	0.398**	0.204	0.187	0.330**	0.389**	1								
8. PSQI global score	5.29 ± 2.84	0.769**	0.763**	0.396**	0.259*	0.535**	0.454**	0.774**	1							
9. BDI-II scores	8.26 ± 8.61	0.555**	0.383**	0.191	0.147	0.372**	0.288*	0.663**	0.617**	1						
10. SDNN	116.21 ± 30.99	−0.047	−0.117	−0.053	0.064	−0.317**	−0.044	−0.131	−0.135	−0.108	1					
11. RMSSD	63.86 ± 24.83	−0.003	−0.006	0.063	−0.044	−0.101	−0.065	−0.046	−0.003	−0.032	0.631**	1				
12. pNN50	37.90 ± 16.44	0.000	0.069	0.063	−0.047	−0.062	−0.069	0.044	0.065	0.014	0.504**	0.917**	1			
13. LF	1312.82 ± 863.15	0.008	−0.038	0.039	−0.037	−0.215	0.059	−0.016	−0.025	0.035	0.758**	0.673**	0.556**	1		
14. HF	1614.01 ± 1320.89	−0.019	0.055	0.029	−0.023	−0.004	−0.075	0.007	0.052	0.025	0.522**	0.923**	0.892**	0.597**	1	
15. LF/HF	1.08 ± 0.72	0.048	−0.045	−0.060	−0.026	−0.201	0.072	0.015	−0.052	0.014	0.067	−0.484**	−0.574**	0.200	−0.564**	1

* *p < 0.05*,

** *p < 0.01*.

## Discussion

This study found that (1) Correlation analysis found that the PSQI global scores was positively correlated with the BDI-II scores, that is, the lower the sleep quality, the higher the level of depressive symptoms. (2) Correlation analysis of PSQI components with 24-h HRV showed that higher LF during awake period is associated with higher subjective sleep quality (i.e., fewer sleep disturbances), and higher SDNN during awake period and bedtime period is associated with higher subjective sleep quality (i.e., fewer sleep disturbances). (3) 41.7% medical students had poor sleep quality, accompanied by relatively high levels of depressive symptoms, less positive emotions, and more negative emotions, which may be related to their heavy academic burden. (4) There was no statistical difference in 24-h HRV indicators between the low PSQI group and the high PSQI group in both awake period and bedtime period. These results suggest that sleep problems are common among medical students, that sleep quality is affected by depressive symptoms, and that cardiac autonomic control is closely related to sleep quality during wakefulness and sleep.

The results showed that the high quality of sleep (low PSQI global scores) group had lower levels of depressive symptoms and reported more positive emotions than the low quality of sleep (high PSQI global scores) group. It may be that the improvement or decrease of sleep experience has a direct impact on the individual's emotional experience, leading to an increase or decrease in the level of depressive symptoms. Studies have pointed out that depression patients experience less positive emotions and more negative emotions (47). Sleep disorders are closely related with depressive symptoms, and sleep insufficiency can lead to depressive symptoms (48). In this way, the individual will enter in a cycle that sleep quality decrease will lead to rising level of depressive symptoms and even for the onset of depression, in turn, the elevated level of depressive symptoms or the onset of depression will lead to the individual in a relatively long period of time remain depressed, thus affecting the quality of sleep. Therefore, if sleep problems do not improve, it can lead to increased levels of depressive symptoms and the onset of depression.

In addition, our results also show that there was a significant positive correlation between PSQI global scores and BDI-II scores. This is consistent with previous research (34). Sleep problems such as primary insomnia, shorten sleep duration, and hypersomnia have been linked to depression and its severity (35, 36, 49–52). These findings further support that individuals with poorer sleep quality have higher levels of depressive symptoms, which may be an important factor in reducing the success rate of treatment for depression, and there may be a complex bidirectional relationship between sleep problems and depression (35, 36). Therefore, more studies are needed to confirm whether the higher risk of depression is caused by sleep problems or the decreased sleep quality caused by the existence of depression, or the simultaneous occurrence of depression and sleep problems. Compared to people with depression, sleep problems, and the application of HRV are relatively easy to study in the general population, and future research could include the study of the biological factors to clarify the mechanisms of the link between depression, sleep quality and HRV.

Contrary to our hypothesis, the 24-h HRV indicators in this study failed to reflect the differences between groups of sleep quality of medical students. This result is in contrast to previous studies which found that SDNN, RMSSD, pNN50, as well as LF and HF were reduced in people with sleep problems compared with controls (10). The differences between these results may be caused by the following reasons. First, the sample size of our study may be small, with a total of 76 participants recruited. After excluding four participants who did not meet the inclusion criteria of the study, 72 valid data were obtained. However, compared with previous studies, the sample size of our study was relatively large, and we did not only recruit female participants (17). Secondly, the samples used in our study were not clinically diagnosed, and the diagnosis of sleep quality depends on self-report. However, for this study, PSQI has a good reliability (Cronbach' s *a* = 0.75), so it has little influence on the results of our study. Finally, the application of 24-h HRV may be more sensitive in clinical samples (4, 10). Even though our study samples met the criteria for grouping sleep quality statistically, no significant statistical difference was found after comparing various indicators of 24-h HRV between the two groups, which needs to be verified again in the comparison between large clinical and non-clinical samples in the future. Furthermore, stress and age might have an impact on an individual's sleep quality and depressive symptoms (53, 54). Individual stress levels were shown to be inversely connected to sleep quality, and depression was a prevalent condition in later life, which has been linked to disability and poor health outcomes over time. Future study should also focus on the role of these elements.

Alternatively, our study results provide a different finding to previous studies. There is a significant negative correlation between SDNN and LF during awake period and the dimension of PSQI, sleep disturbance(*r*_*SDNN*_ = −0.285, *r*_*LF*_ = −0.239, *p* < 0.05), but the former two are not associated with depressive symptoms. We found a significant negative correlation between SDNN during bedtime period and the dimension of PSQI, sleep disturbance (*r* = −0.317, *p* < 0.01). SDNN reflects overall HRV and RMSSD as well as pNN50 reflect parasympathetic activity. Firstly, this indicates that the sleep disturbance in PSQI components can more sensitively reflect the sleep quality of medical students, and individuals with higher sleep disturbance may have lower SDNN in awake period and bedtime period, while LF in awake period is lower. Future research would need to replicate the results using larger samples, and the samples expanded to include different demographic groups so as to further analyze the sources of sleep quality problems and HRV characteristics of medical students. Provide targeted guidance for follow-up interventions. Second, the LF reflects a mixture of parasympathetic and sympathetic contributions (55) with greater sympathetic sensitivity (56). The HF is thought to reflect the variation in heart rate during the respiratory cycle (also referred to as respiratory sinus arrhythmia), and it has been suggested to be almost exclusively dependent on the parasympathetic activity, that is, with reductions in HF measures of HRV, we are assessing a reduction in vagal tone (57). Low frequency components and HF measures can be assessed using both long recordings (24 h) and short recordings (5 min). According to the results of our study, the sleep quality is related to the overall HRV. Low frequency components and SDNN druing awake period can jointly reflect the sleep quality, while SDNN during sleeping period can reflect sleep quality. The sympathetic nervous system is more sensitive to behavior that is uncertain, novel and threatening, and this response is often referred to as the sympathetic fight-or-flight preparation for action (58). This response to threat may be related to the well-known negative bias, in which negative information takes precedence over positive information in guiding behavior (59). Hence, LF, as an indicator of sympathetic nervous system, can sensitively reflect individual sleep quality. Although the balance of sympathetic and parasympathetic nervous system is the basis of maintaining human physiological function, the sympathetic activity is dominant during daytime arousal, while parasympathetic activity is dominant during night sleep, and the two are in an antagonistic and balanced state at any time (60, 61). Our results suggest that sleep should be mediated by both the sympathetic and parasympathetic nervous systems, rather than solely by the parasympathetic nervous system.

In any case, the relationship between sleep problems and depressive symptoms/depression is consistent with previous findings, but the relationship between 24-h HRV and the former two or the use of waking and sleeping HRV indicators as indicators of sleep quality, depressive symptoms/depression needs further verification. Studies of sympathetic and parasympathetic nerves have shown that autonomic imbalances are precursors to disease development and other health-related risks (62–64). Thus, in order to improve the sleep quality of college students, in addition to enhance the pertinence and effectiveness of mental health education, psychological census should be conducted after the students enter school and psychological files should be established for them. The present study results suggested that the mindfulness intervention is effective for positive results of mental health, it can also reduce the physical symptoms associated with anxiety, self-perception stress levels, and relieve the symptoms of mental illness severity.

The limitations of this study are as follow. Firstly, this is a cross-sectional study, so it is still not know how is the dynamic relationship among depressive symptom, sleep quality and 24-h HRV. Future study need to address this issue. Second, depressive symptom and sleep quality are all assessed through a single source of self-report questionnaire. Relying on this subjective method can, therefore, be problematic. In future research, self-report approaches should be combined with clinical diagnosis and objective measures. Lastly, the participants surveyed in the current study were all medical students. Therefore, they are not sufficiently representative of the general population, and the extrapolation validity of this research is limited. Future study might focus on senior medical students as research subjects, or on medical students of all grades as research subjects for comparative studies. Simultaneously, data on recent stress levels, the time living under pressure, and resilience need to be gathered in order to further investigate and explain the association between sleep quality and 24-h HRV in medical students.

In conclusion, we found that sleep problems were more common among medical students and were associated with higher levels of depressive symptoms and more negative emotional experiences. Meanwhile, the results of our study provide supplementary explanation for the past research, we should not only pay attention to the relationship between HRV and sleep quality in the sleep stage, but also pay attention to the relationship between HRV and sleep quality in the wake stage. As a complex and comprehensive physiological process, sleep may not only rely on the role of parasympathetic nerves. The sympathetic or the whole autonomic nervous system also plays an indispensable coordinating role in sleep. This not only reminds us to pay more attention to the physical and mental health problems of medical students, but also to formulate targeted and effective prevention and intervention measures. Heart rate variability during awake and bedtime period may become effective applications for individual physical and mental health in the future. Future studies can further investigate the application value, sensitivity and specificity of 24-h HRV analysis in a large sample of normal population or a large clinical sample, so as to clarify the relationship between sleep quality, depressive symptoms and HRV indices. In terms of measuring instruments, future research may examine the differences in measurement outcomes between the patch dynamic electrocardiograph employed in this study and the smart wrist watch.

## Data Availability Statement

The raw data supporting the conclusions of this article will be made available by the authors, without undue reservation.

## Ethics Statement

The studies involving human participants were reviewed and approved by Academic Ethics Committee of Southern Medical University. The patients/participants provided their written informed consent to participate in this study.

## Author Contributions

XG, TS, and HX collected and analyzed the data. XG and RX interpreted the data and wrote the first draft of the manuscript. ZX and XG generated the idea, designed the study, and wrote the manuscript. All authors contributed to the article and approved the submitted version.

## Funding

This study was supported by grants from the National Natural Science Foundation of China to ZX (32070994, 31872769).

## Conflict of Interest

The authors declare that the research was conducted in the absence of any commercial or financial relationships that could be construed as a potential conflict of interest.

## Publisher's Note

All claims expressed in this article are solely those of the authors and do not necessarily represent those of their affiliated organizations, or those of the publisher, the editors and the reviewers. Any product that may be evaluated in this article, or claim that may be made by its manufacturer, is not guaranteed or endorsed by the publisher.
